# Antibacterial Activity of *Crocus sativus* L. Petals Extracts against Foodborne Pathogenic and Spoilage Microorganisms, with a Special Focus on *Clostridia*

**DOI:** 10.3390/life13010060

**Published:** 2022-12-25

**Authors:** Sara Primavilla, Cinzia Pagano, Rossana Roila, Raffaella Branciari, David Ranucci, Andrea Valiani, Maurizio Ricci, Luana Perioli

**Affiliations:** 1 Istituto Zooprofilattico Sperimentale dell’Umbria e delle Marche “Togo Rosati”, Via Salvemini 1, 06126 Perugia, Italy; 2 Department of Pharmaceutical Sciences, University of Perugia, Via del Liceo 1, 06123 Perugia, Italy; 3 Department of Veterinary Medicine, University of Perugia, Via San Costanzo 4, 06121 Perugia, Italy

**Keywords:** saffron petals, natural antibacterial, agar well-diffusion, MIC, MBC, time-kill test, *Clostridium* spp., kinetic parameters, food safety, by-product reuse

## Abstract

In recent years, there has been a growing interest in the use of novel antimicrobial agents able to inhibit or kill food-borne bacteria or to interrupt the onset of food spoilage. *Crocus sativus L.* petals, typically considered as waste obtained from saffron spice production, could be a source of natural bioactive compounds to be used as food preservatives. The purpose of this work was to investigate the antibacterial properties of two hydroalcoholicsaffron petal extracts obtained by maceration (SPEA) and by ultrasonic bath (SPEB) methods. The main polyphenols identified in both extracts were gallic and chlorogenic acids, representing almost 70% of the phenolic fraction monitored. The antibacterial activity was studied by the agar well-diffusion method, against food-borne pathogenic and spoilage bacteria. Both extracts showed activity mainly against Gram-positive bacteria, in particular those belonging to the *Clostridiaceae* family (*C. perfringens*, *C. botulinum* and *C. difficile*), with inhibition zone diameters ranging from 13 to 18 mm. The antibacterial properties against *Clostridia* were further analyzed, determining MIC and MBC and performing a time-kill test. SPEA showed lower MIC/MBC values (250 mg/mL) compared to SPEB (500 mg/mL), suggesting that it could be more active against the assayed strains, probably because of its higher content of gallic acid. SPEA and SPEB, tested at a concentration of 1 × MIC, showed bactericidal activity against *C. perfringens*, *C. botulinum* and *C. difficile* and these results suggest that saffron petals could represent a valuable natural alternative source to conventional preservatives. Further investigations are needed to evaluate possible applications in the food industry.

## 1. Introduction

Food microbiological contamination by food-borne pathogenic bacteria represents a critical concern to both consumers and the food industry worldwide [1]. Particularly, according to European guidelines 2021 European Center for Disease Control (ECDC)—European Food Safety Authority (EFSA) summary report on zoonoses, during 2019–2020, 8261 food-borne outbreaks involving 69,480 cases of illness, 5534 hospitalisations and 94 deaths were reported. These data may be slightly underestimated due to the indirect consequences of the COVID-19 pandemic among EU populations in 2020 leading to a reduced exposure of people to contaminated food and a higher under-reporting of outbreaks [2,3].

In order to enhance the safety and increase the shelf-life of food products, many food preservation strategies, including chemical antimicrobials, have been traditionally used at an industrial level to control the microbial growth in foods [4]. In recent times, the increased awareness of the impact of the diet on human health has encouraged the scientific community and food industries to search for effective alternatives to the chemical antimicrobial additives commonly used in food preservation [5]. The use of these compounds indeed, although strictly regulated [6], is considered with mistrust by consumers, due to their potential long-term adverse effects on health [7,8]. Some of the main synthetic antimicrobials, approved by regulatory agencies, are: sodium benzoates and propionates, potassium sorbates, sorbic acid, sulphites, chlorides, nitrites, triclosan, nisin, natamycin, potassium lactate, ascorbic acid, citric acid, tartaric acid, etc. [9]. Gutiérrez-del-Río et al. reported some examples of synthetic approved antimicrobials commonly used in the food industry, which can represent a health threat for the consumer. Sulfites, for instance, have been associated with some anti-nutritional consequences such as the degradation of thiamine or vitamin B1 in food [10].

In this context, bioactive compounds deriving from natural matrices, especially agro-industrial wastes, are gaining attention as potential alternative sources of food preservatives [11,12]. Fruit and vegetable processing, indeed, originates large amounts of wastes and their possible reuse in the food industry could have positive effects both from the environmental and economic point of view [13]. Furthermore, food safety could benefit from the application of these bioactive compounds in food production due to their ability to inhibit food-borne bacteria and food spoilage [14,15].

This aspect is of utmost importance related to the notable increase in the number of antibiotic-resistant pathogens registered in recent decades [16]. The selective pressure deriving from the use of antibiotics in primary production and of biocides, such as disinfectants, decontaminants or food and feed preservatives, contributes to the spreading of antimicrobial resistance throughout the food chain [17]. Natural and bio-based antibacterial agents could represent an important valuable alternative as they are considered to evade the growing resistance of some pathogens, with the assumption that bacteria have less chance of developing resistance to natural antibacterials [18]. 

*Crocus sativus Linnaeus*, commonly known as saffron, is known mainly for the stigma, its noble part rich in active compounds, used as a food spice, flavoring and preserving agent and as a raw material for health products and cosmetics [19,20,21,22]. However, very little research has been performed on the petals [23], which represent the main by-product of the spice production, where more than 90% of the plant material is discarded [24]. In compliance with the principles of a circular economy, it would be of the utmost importance to find valuable alternatives to utilize saffron flower waste, in particular petals that could be good sources of bioactive compounds, such as flavonol glucosides, flavonoid glycosides, crocin, kaempferol, anthocyanins and lutein diester [24]. Previous studies clearly suggest that saffron petals can be used to obtain extracts with different properties such as antioxidant activity and, in particular, antibacterial activity against some Gram-positive and Gram-negative bacteria and fungi. [25,26]. For instance, Asgarpanaha et al. described a significant antibacterial activity of *C. sativus* petals methanolic extract against *Staphylococcus aureus*, *Bacillus cereus*, *Salmonella* Typhi, *Escherichia coli* and *Shigella dysenteriae* [27]. Wali et al., observed the antibacterial activity of three saffron petal extracts obtained by solvents of different polarity reporting good results for *P. aeruginosa* and *S. aureus* [28].

As the behavior of the extracts changes according to the extraction solvent as well as the extraction procedure, the purpose of this work is to thoroughly characterize the in vitro antibacterial properties of two different saffron petal extracts produced by eco-friendly methods (maceration and ultrasonic bath) using ethanol as the extraction solvent [29]. The attention was particularly focused on micro-organisms related to consumers’ health, such as food-borne pathogens and food spoilage bacteria. 

## 2. Materials and Methods

### 2.1. Extract Preparation and Determination of the Phenolic Profile

Saffron petals used for extract preparation were hand-harvested in October 2019 and provided by the farm “*UBI MAIOR*” (Roccasalli, Rieti, Italy). After harvesting, saffron petals were immediately frozen in order to maintain their original features. Then, the obtained extracts were freeze-dried and stored under CaCl2 (as the lyophilized products are sensitive to humidity) until their use.

Two different dry extracts were prepared, according to the procedure described by Pagano et al. [29], using freeze-dried saffron petals/extraction solvent in a ratio of 2.46 g/200 mL:

- saffron petal extract A (SPEA), was obtained by the maceration method using ethanol (EtOH-Sigma-Aldrich, Milan, Italy) 70% at 45 °C under magnetic stirring (400 rpm) for 90 min (extraction yield 58.94 ± 0.05 g/100 g of freeze-dried petals);
- saffron petal extract B (SPEB) was obtained by suspending the freeze-dried petals in using EtOH 70% under an ultrasonic bath (BANDELIN, RK 100H, frequency 35 kHz, 80/320 W) for 10 min, then left in static conditions at room temperature (25 °C) overnight (extraction yield 56.55 ± 3.74 g/100 g of freeze-dried petals).

After the extraction time, in both cases, the solvent was separated from the exhausted petals by filtration under vacuum using filter paper and then concentrated by rotary evaporator (Büchi, R-100, Cornaredo, Italy) at a working temperature of 35 °C in order to maintain the original features of the extracted bioactives. The concentrated product was diluted in 25 mL of bidistilled water, freeze-dried and stored under CaCl_2_ until use. 

The phenolic qualitative and quantitative profile of the extracts was defined as described in a previous study [29], using the method originally developed by Simeoni et al. [30] properly modified. The chromatographic analysis was performed by a Nexera XR UHPLC system (Shimadzu, Tokyo, Japan) equipped with an ACE Excel 2 C18-PFP column (10 cm × 2.1 mm id; ACE, Aberdeen, UK) packed with particles of 2 μm. Compound identification was performed through a QTRAP 4500 tandem mass spectrometer (Sciex, Toronto, ON, Canada), coupled with an electro-spray ionization source (V-source) operating in negative ionization mode.

### 2.2. In Vitro Evaluation of Antibacterial Activity—Agar Well-Diffusion

SPEA and SPEB antibacterial activity were determined by the agar well-diffusion method against different food-borne pathogenic and spoilage bacteria. All the tested strains were bought from Microbiologics, St. Cloud, MN, USA, except for *C. botulinum* which was provided by the Italian National Institute of Health (National Referral Center for Botulism). 

As previously described [31,32], for each organism a suspension of 0.5 McFarland in 0.9% sterile saline solution was prepared. Then, 100 µL were distributed on each quadrant of Mueller-Hinton agar (MHA)/Mueller-Hinton agar 5% defibrinated sheep blood (MHAB) plates (Oxoid Limited, Basingstoke, UK) by a swab. Holes of 7 mm diameter were made in the plates, by scooping out medium with a sterilized cork borer and 50 µL of extract solution in sterile demineralized water (1000 mg/mL) were inoculated. 

The plates were incubated according to the growth conditions shown in Table 1 and for each bacterial strain, SPEA and SPEB were tested. Negative control was set up with the same solvent used to prepare the extract solution (sterile demineralized water), while antibiotic discs (Oxoid Limited, Basingstoke, UK) were used as positive control (Table 1). At the end of the incubation time, the presence and the diameter of the inhibition halo was evaluated by a gauge (mm). The measurements were performed in triplicate to determine the mean of the inhibition zone and standard deviations were calculated.

### 2.3. Minimal Inhibitory Concentration (MIC) and Minimal Bactericidal Concentration (MBC) Determination

To further investigate the antibacterial activity of SPEA and SPEB, their MICs/MBCs were determined on *C. perfringens*, *C. botulinum* and *C. difficile*. These three micro-organisms were chosen considering the results of the agar well diffusion test. MICs/MBCs were measured using a standard broth microdilution technique, according to Clinical Laboratory Standards Institute (CLSI) guidelines [33]. The bacterial suspensions used for the assay were prepared adjusting the number of bacteria to 10^5^ CFU/mL with fresh Mueller-Hinton broth with 5% blood (Biolife Italiana s.r.l., Milan, Italy). Aliquots of each suspension were added to 96-well microplates (Starlab International GmbH, Hamburg, Germany) containing the same volumes of two-fold serial dilution (ranging from 1 to 0.0078 g/mL) of the extracts. Moreover, three controls were set up: these included antibiotic control (with benzylpenicillin sodium salt; Sigma-Aldrich, St. Louis, MO, USA), organism control (with culture medium and bacterial suspension) and negative control (with culture broth and the extract solution at the same concentration tested). The plates were incubated for 48 h at 37 °C under anaerobic conditions (anaerobic jar—2.5 L AnaeroJar, AG002, with AnaeroGen 2.5 L, AN0025, Oxoid Limited, Basingstoke, UK). MIC was defined as the lowest concentration of extract that produced no bacterial growth when compared to time 0 wells [34]. The MBC was determined by subculturing the broths used for MIC determination. A quantity of 10 μL of broths culture of the wells, corresponding to the MIC and to the higher MIC concentrations, was plated onto fresh 5% Sheep Blood agar dishes (Microbiol s.r.l., Cagliari, Italy) and then incubated for 48 h at 37 °C, under anaerobic conditions. The MBC was represented as the smallest amount of extract that was capable of killing the bacterial inoculum, demonstrated by the total absence of growth [34]. All tests were performed in triplicate and the results were expressed as means ± standard deviation.

### 2.4. Time-Kill Test and Evaluation of Growth Dynamics

The time-kill test was performed in order to characterize the bactericidal activity of the extracts against *C. perfringens*, *C. botulinum* and *C. difficile*. The bactericidal effect is defined as a decrease of at least 3 Logs or the killing of 99.9% of viable cells in a specific time [35]. As described in the CLSI guidelines—document M26-A [33], three broth cultures were set up in Mueller-Hinton broth with 5% blood containing a bacterial concentration of 10^5^ CFU/mL. The two extracts (SPEA, SPEB) were added respectively to the first two broth cultures, in order to have a final concentration of 1 x MIC. The last broth culture was used as a growth control (CTRL). The broth cultures were incubated at 37 °C under anaerobic conditions and at various time intervals (0-2-4-6-8-12-24 and 48 h) and the viable cells (CFU/mL) were counted. All time-kill curve experiments were performed in triplicate; the results were expressed as Log of viable cell numbers (Log CFU/mL) and standard deviations were calculated. 

*C. perfringens*, *C. botulinum* and *C. difficile* growth curves for SPEA, SPEB and CTRL broth cultures were defined using DMFit version 2021 (ComBase online freeware) by fitting the experimental data to the model of Baranyi and Roberts [36]. From the resultant curves, the initial values (Log CFU/mL), duration of the lag phase (λ) in h, maximum growth rate (μmax) (Log CFU/mL/h) and final values (Log CFU/mL) were reported. When bacteria were not detected at the 1.00 Log CFU/mL level (<10 CFU/mL), a value of −0.50 Log CFU/mL was assigned [34].

### 2.5. Statistical Analysis

The data obtained from the agar well-diffusion and time kill-test were statistically analyzed by an analysis of variance (ANOVA) model, using the generalized linear model (GLM) procedure of SAS (SAS Institute Inc., Cary, NC, USA, 2001). For the first assay a mixed model was used with treatments (A, B and CTRL) and micro-organisms as fixed effects. For the second test the same model was used with treatments (A, B and CTRL) and time (T0, T2, T4, T6, T8, T12, T24, T48) as fixed effects. The replicate effect was found not significant and removed from the model. To explain significant mean differences (*p* < 0.05), Tukey’s post-hoc analysis was performed.

The effects of formulation on the growth of the target micro-organisms were evaluated with the DMFit tool of the free predictive microbiology software Combase (https://www.combase.cc/index.php/en/DMFit, accessed on 20 July 2022), allowing for the definition of growth parameters such as lag phase duration (1/h) and maximum growth rate (max, 1/h) by means of the Baranyi and Roberts model [36]. The fitted results were analyzed by a one-way ANOVA model with treatment as fixed effect and Tukey’s test (*p* < 0.05).

## 3. Results and Discussion

### 3.1. Phenolic Profile of the Extracts

**Figure 1:**
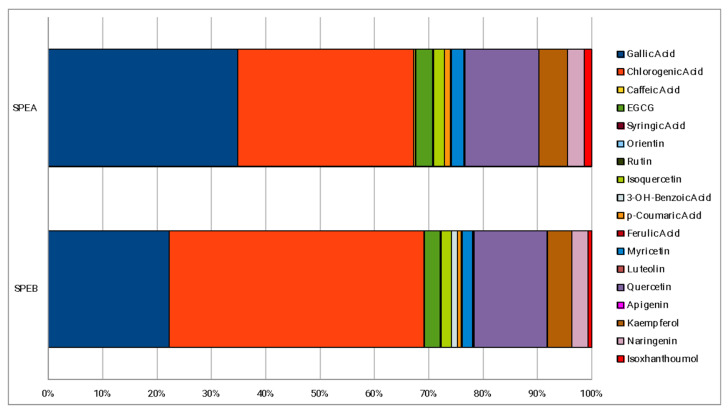
Phenolic percentage composition of the extracts.

The percentage composition of 18 phenolic compounds, identified in a previous work [29], is reported in Figure 1. The main polyphenols detected in both extracts were gallic and chlorogenic acids, representing almost 70% of the phenolic fraction monitored. Quercetin and kaempferol, represent the other two polyphenols most present in both SPEA and SPEB. The main difference between SPEA and SPEB is the higher content of gallic acid of SPEA (34.84%) compared to SPEB (22.22%), which, instead, is richer in chlorogenic acid (46.81%) compared to SPEA (32.36%). With regards to the other phenolic compounds, no particular differences about the percentage composition were found.

### 3.2. In Vitro Evaluation of Antibacterial Activity—Agar Well-Diffusion

The in vitro antibacterial activity of SPEA and SPEB against the selected bacteria was qualitatively and quantitatively assessed from the presence or absence of inhibition zones. A preliminary screening test was performed by the agar well-diffusion method. Table 2 reported the inhibition halos (mm) measured for each micro-organism. 

SPEA and SPEB (1000 mg/mL) showed comparable results with each other, with a significant antibacterial activity against the Gram-positive bacteria tested, with an inhibition zone diameter ranging from 11 to 18 mm. Rare or totally absent antibacterial activity was observed, instead, against Gram-negative bacteria involved in the study. Similar results were observed in a previous study, where a methanolic saffron petal extract was tested against Gram-positive and Gram-negative pathogenic bacteria [25]. The greater susceptibility of Gram-positive compared to Gram-negative bacteria can be explained by the structural differences in their cell walls, which lead to a higher vulnerability to membrane permeability alteration of Gram-positive than Gram-negative [37]. The chemical analysis reported in a previous work [29] and in Figure 1 showed that both the extracts are rich in gallic and chlorogenic acids. Several studies investigated the antimicrobial properties of these two phenolic compounds. With regards to chlorogenic acid, antimicrobial activity was observed against a wide range of organisms, including Gram-positive and Gram-negative bacteria, yeasts, molds, viruses, and amoebas [38]. In particular, Luís et al. observed the anti-staphylococcal properties of both gallic and chlorogenic acids and their ability to influence the adhesion properties of *S. aureus* and, thus, to contrast the biofilm formation [39]. The antimicrobial properties of gallic acid could be attributable to its ability to destroy the structural integrity of bacteria [40] or inhibit the formation of bacterial biofilm in vitro [41]. In the case of chlorogenic acid, because of its negative surface charge, it can be hypothesized that it might bind to the cell membrane, leading to the loss of the barrier function or blocking the nutrient flow [42].

**Figure 2:**
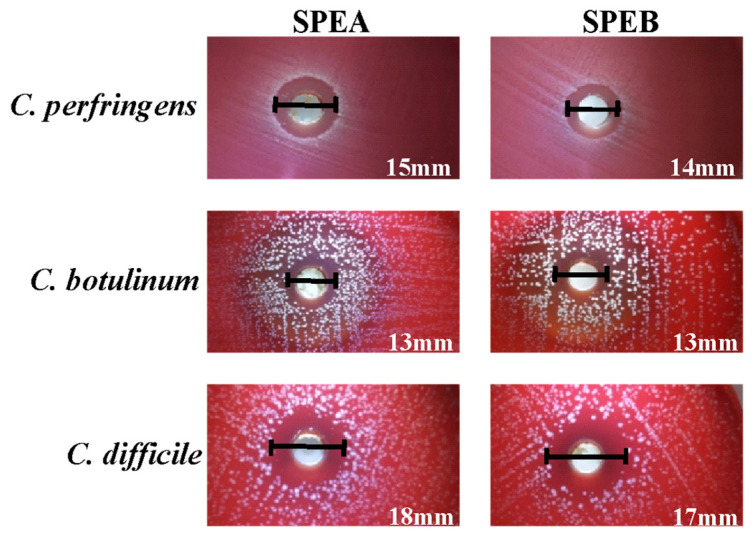
Inhibition halos obtained for *C. perfringens*, *C. botulinum* and *C. difficile* in the screening test.

Among the Gram-positive bacteria, both extracts were particularly active against the strains belonging to the *Clostridiaceae* family (halo diameters between 13 and 18 mm) (Figure 2).

These results are supported by literature data, where the anti-clostridial effect of different phenolic compounds was observed. Hamad et al. demonstrated that probiotic cell-free supernatants, particularly rich in gallic and chlorogenic acid, can exhibit inhibitory activity against *C. botulinum* type E [43]. Lee et al. observed that gallic acid derived from tea extracts decreases the levels of *C. perfringens*, *C. difficile* and *Bacteroides* spp. observing an inhibitory effect selective against pathogenic *Clostridium* spp. [44].

Strains belonging to the *Clostridiaceae* family are of considerable importance in the food industry, since *C. perfringens* can be involved in food poisoning, due to the ingestion of contaminated food and the enterotoxin is responsible for the development of symptoms (abdominal cramps, diarrhea, nausea and vomit) [45]. *C. botulinum* is responsible for food-borne poisoning botulism, with high mortality rates in both animals and humans [46]. *C. difficile*, instead, has been considered for years one of the main causes of nosocomial diarrhea in hospitalized patients after antibiotic treatment. The increasing incidence, recently recorded, has led to the investigation of other possible sources of *C. difficile* acquisition, including the ingestion of contaminated food [47].

### 3.3. Determination of MIC and MBC

Based on this information, it was interesting to further investigate the effect of the two extracts against *C. perfringens*, *C. botulinum* and *C. difficile* by the determination of MIC and MBC. The obtained results, reported in Table 3, highlight lower MIC/MBC values for SPEA (250 mg/mL) compared to SPEB (500 mg/mL), suggesting that it could be more active against the assayed strains. The MIC/MBC obtained in the present study are higher than those reported by some authors in literature for natural compounds [48]. On the other hand, the present results are in agreement with some other studies showing similar levels of MIC and MBC, albeit for other bacteria [49]. The efficacy of a natural extract against microbial growth is strongly related to its chemical composition and the extraction technique. Therefore, it can be hypothesized that the definition of an extraction technique able to concentrate the bioactive compounds, enhancing the extract’s efficacy, would help to define its most suitable application.

As already discussed, it was observed that SPEA contains a higher amount of gallic acid compared to SPEB, suggesting that the stronger antibacterial activity observed could be mainly attributed to this molecule. Moreover, it is possible to hypothesize that the other molecules present in SPEA may have synergistic effects. For example, literature data demonstrated the activity of isoxanthohumol (2.43 µg/g in SPEA and 1.29 µg/g in SPEB [25]) against *C. perfringens* and *C. difficile* [50]. Further studies are needed to demonstrate the effective contribution of each identified molecule, especially considering the possibility that the observed activity may be attributable to the phytocomplex rather than to the individual compounds. 

### 3.4. Time-Kill Test and Evaluation of C. perfringens, C. botulinum and C. difficile Growth Dynamics

The results of the time-kill test are reported in Table 4. Both extracts showed a strong bactericidal activity against *C. perfringens*, *C. botulinum* and *C. difficile* hindering the growth of these micro-organisms since the first few hours and then reducing the initial values of more than 3 Log CFU/mL at the end of the time frame considered (from 4.76–4.04–4.58 for *C. perfringens*, *C. botulinum* and *C. difficile,* respectively, to −0.50 Log CFU/mL at 48 h) (Table 4). Data describe that the broth cultures treated with SPEA and SPEB evolved in a similar way for the three tested strains. Starting from T6, SPEA and SPEB began to record values that were statistically lower than CTRL and from T8, SPEB registered slightly lower values than SPEA until T24 where in both cultures there was no more growth.

**Figure 3:**
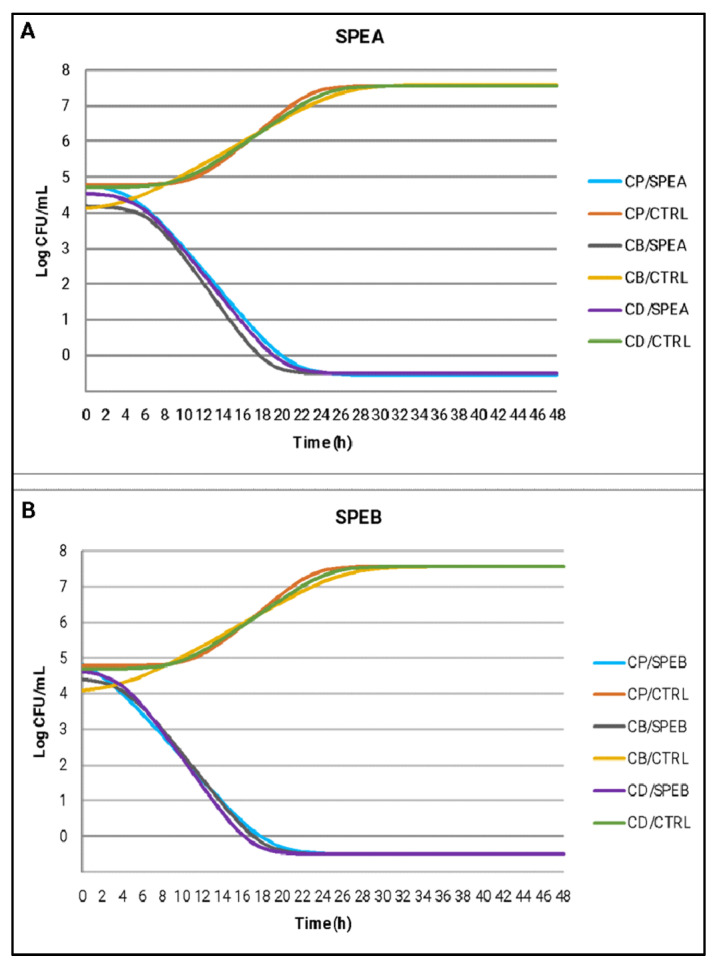
Estimated growth curves of *C. perfringens* (CP), *C. botulinum* (CB) and *C. difficile* (CD) using the Baranyi and Roberts model, in broth cultures treated with SPEA (**A**), SPEB (**B**) compared to CTRL samples.

The same observations can be made by analyzing the estimated growth curves of *C. perfringens*, *C. botulinum* and *C. difficile* under the effect of SPEA and SPEB compared to CTRL (Figure 3). The curves represent the best fitting for the raw growth data over time of incubation and Table 5 reports the growth kinetic parameters obtained by modeling. SPEB recorded for the three tested micro-organisms a slightly reduced λ phase compared to SPEA (average values of 2.36 h) and for this reason, SPEB broth culture reached the final value about 2 h earlier than the SPEA broth culture. The literature lacks studies investigating the effects of natural compounds against food-related *Clostridiaceae*, in particular no studies on bacterial death kinetics after exposure to saffron petal extracts are available, hindering a thorough comparison of the results.

## 4. Conclusions

This research showed the antibacterial (bactericidal) properties of saffron petal extracts obtained using EtOH 70% as the extraction solvent and maceration or ultrasonic bath as extraction methods. The extracts were particularly active against strains belonging to the *Clostridiaceae* family (*C. perfringens*, *C. botulinum* and *C. difficile*), with similar results recorded. SPEA showed lower MIC/MBC values (250 mg/mL) compared to SPEB (500 mg/mL), showing that it is more active against the assayed strains. These results suggest that, despite the relatively high MIC/MBC values, saffron petals could representa valuable source of active ingredients, to be used as alternatives to conventional preservatives, especially in consideration of the green and eco-friendly nature of the extracts. Future studies are needed to evaluate possible applications in the food industry, aiming to protect consumers’ health with a sustainable approach. Of the utmost importance would be to test these compounds against multi-drug resistant bacteria to define an alternative tool to face the growing issue of drug resistance. Moreover, the extracts can find applications as preservatives in other fields such as health (e.g., cosmetics, medicines, etc.).

## Figures and Tables

**Table 1:** Micro-organisms, growth conditions and positive controls used for the agar well-diffusion test.

Microorganisms		Growth Conditions	Positive Controls
Gram+ bacteria	*Staphylococcus aureus* WDCM 00034	37 °C—24 h in MHA	Tetracycline 30 µg/disc
	*Bacillus cereus* WDCM 00001	30 °C—24 h in MHA	Penicillin G 10 UI/disc
	*Enterococcus faecalis* WDCM 00087	37 °C—24 h in MHA	Tetracycline 30 µg/disc
	*Listeria monocytogenes* WDCM 00021	37 °C—24 h in MHA	Tetracycline 30 µg/disc
	*Clostridium perfringens* WDCM 00007	37 °C—24–48 h under anaerobic conditions in MHAB	Penicillin G 10 UI/disc
	*Clostridium botulinum* ISS CNRB CL 14NT	37 °C per 24–48 h under anaerobic conditions in MHAB	Penicillin G 10 UI/disc
	*Clostridioides difficile* CDC 20120296	37 °C—24–48 h under anaerobic conditions in MHAB	Penicillin G 10 UI/disc
Gram− bacteria	*Pseudomonas aeruginosa* WDCM 00025	25 °C—24–48 h in MHA	Gentamycin 10 µg/disc
	*Salmonella* Enteritidis WDCM 00097	37 °C—24 h in MHA	Tetracycline 30 µg/disc
	*Escherichia coli* WDCM 00013	37 °C—24 h in MHA	Tetracycline 30 µg/disc
	*Campylobacter jejuni* WDCM 00005	41.5 °C—48 h under microaerobic conditions in MHAB	Erythromycin 15 µg/disc

**Table 2:** Inhibition halos obtained for SPEA and SPEB for the different strains tested. Values are expressed as means ± standard deviation.

Microorganisms		Diameter of the Inhibition Halo (mm)		
		SPEA	SPEB	Positive Control
Gram+ bacteria	*Staphylococcus aureus*	11.33 ± 0.58 aA	12.33 ± 0.58 aA	21.00 ± 1.00 bB
	*Bacillus cereus*	11.33 ± 0.58 aA	12.33 ± 0.58 aB	15.67 ± 1.15 aC
	*Enterococcus faecalis*	-	-	21.67 ± 0.58 b
	*Listeria monocytogenes*	10.67 ± 0.58 aA	11.00 ± 1.00 aA	20.33 ± 1.15 bB
	*Clostridium perfringens*	14.33 ± 0.58 bA	14.00 ± 1.00 bA	30.67 ± 0.58 dB
	*Clostridium botulinum*	14.00 ± 1.00 bA	13.67 ± 0.58 bA	33.00 ± 1.00 dB
	*Clostridioides difficile*	17.67 ± 0.58 cA	18.00 ± 1.00 cA	33.67 ± 1.53 dB
Gram− bacteria	*Pseudomonas aeruginosa*	-	-	18.00 ± 1.00 ab
	*Salmonella* Enteritidis	-	-	17.00 ± 1.00 a
	*Escherichia coli*	-	-	16.33 ± 0.58 a
	*Campylobacter jejuni*	11.67 ± 0.58 aA	11.67 ± 0.58 aA	25.33 ± 1.53 cB

Different letters in the same row (A,B,C) indicate differences between mean values for the two extracts and the control group (*p* < 0.05); different letters in the same column (a,b,c,d) indicate differences between mean values for different micro-organisms (*p* < 0.05). SPEA = saffron petals extract A obtained by maceration under magnetic stirring (400 rpm); SPEB = saffron petals extract B obtained by under ultrasonic bath; positive control = antibiotic discs.

**Table 3:** Minimum inhibitory concentration (MIC) and minimum bactericidal concentration (MBC) against *C. perfringens*, *C. botulinum* and *C. difficile*. Values are expressed as means ± standard deviation.

Microorganism	SPEA (mg/mL)		SPEB (mg/mL)		Benzylpenicillin (µg/mL)	
	MIC	MBC	MIC	MBC	MIC	MBC
*C. perfringens*	250	250	500	500	0.06	0.06
*C. botulinum*	250	500	500	500	0.5	0.5
*C. difficile*	250	250	500	500	1	1

SPEA = saffron petals extract A obtained by maceration under magnetic stirring (400 rpm); SPEB = saffron petals extract B obtained by under ultrasonic bath.

**Table 4:** Results of the time-kill test. Values are expressed as means ± standard deviation.

Time	Treatment	*C. perfringens* (Log CFU/mL)	*C. botulinum* (Log CFU/mL)	*C. difficile* (Log CFU/mL)
T0	CTRL	4.76 ± 0.18 A	4.04 ± 0.05 A	4.58 ± 0.14 A
	SPEA	4.76 ± 0.18 D	4.04 ± 0.05 D	4.58 ± 0.37 D
	SPEB	4.76 ± 0.18 E	4.04 ± 0.05 D	4.58 ± 0.37 E
T2	CTRL	4.59 ± 0.14 A	4.28 ± 0.15 abA	4.59 ± 0.03 A
	SPEA	4.80 ± 0.25 D	4.08 ± 0.06 aD	4.41 ± 0.18 D
	SPEB	4.54 ± 0.17 E	4.49 ± 0.08 bE	4.36 ± 0.11 E
T4	CTRL	4.75 ± 0.21 bA	4.32 ± 0.20 A	4.64 ± 0.10 A
	SPEA	4.59 ± 0.34 bD	4.26 ± 0.17 D	4.36 ± 0.26 D
	SPEB	4.08 ± 0.05 aD	4.46 ± 0.19 E	4.45 ± 0.18 E
T6	CTRL	4.98 ± 0.13 cA	4.40 ± 0.11 bA	4.94 ± 0.08 cAB
	SPEA	4.23 ± 0.27 bD	4.04 ± 0.05 aD	4.23 ± 0.11 bD
	SPEB	3.70 ± 0.27 aD	3.86 ± 0.19 aD	3.90 ± 0.12 aD
T8	CTRL	4.91 ± 0.12 cA	4.72 ± 0.16 cB	4.90 ± 0.07 cAB
	SPEA	3.52 ± 0.32 bC	3.26 ± 0.27 bC	3.46 ± 0.10 bC
	SPEB	2.30 ± 0.10 aC	2.32 ± 0.17 aB	2.45 ± 0.15 C
T12	CTRL	5.08 ± 0.08 cA	5.43 ± 0.17 bC	5.11 ± 0.08 cB
	SPEA	2.26 ± 0.25 bB	2.08 ± 0.11 aB	2.32 ± 0.09 bB
	SPEB	1.80 ± 0.15 aB	1.90 ± 0.07 aB	1.60 ± 0.13 aB
T24	CTRL	7.46 ± 0.09 bB	7.11 ± 0.19 bD	7.32 ± 0.17 bC
	SPEA	−0.50 ± 0.00 aA	−0.50 ± 0.00 aA	−0.50 ± 0.00 aA
	SPEB	−0.50 ± 0.00 aA	−0.50 ± 0.00 aA	−0.50 ± 0.00 aA
T48	CTRL	7.54 ± 0.36 bB	7.56 ± 0.16 bE	7.54 0.28 bC
	SPEA	−0.50 ± 0.00 aA	−0.50 ± 0.00 aA	−0.50 ± 0.00 aA
	SPEB	−0.50 ± 0.00 aA	−0.50 ± 0.00 aA	−0.50 ± 0.00 aA
SEM		0.111	0.077	0.095
*p* value	Time	<0.001	<0.001	<0.001
	Treatment	<0.001	<0.001	<0.001
	T×T	<0.001	<0.001	<0.001

Considering each of the tested micro-organisms, different letters for the same sampling time (a,b,c) or for the same treatment during sampling times (A,B,C,D,E) denote statistical difference (*p* < 0.05). SPEA = saffron petals extract A obtained by maceration under magnetic stirring (400 rpm); SPEB = saffron petals extract B obtained by under ultrasonic bath; CTRL = growth control.

**Table 5:** Estimated growth dynamic parameters using the Baranyi and Roberts model for *C. perfringens*, *C. botulinum* and *C. difficile* growth in broth cultures treated with SPEA and SPEB compared to CTRL samples.

Microorganisms and Parameters	SPEA	SPEB	CTRL
*C. perfringens*			
Initial values (Log CFU/mL)	4.768 ± 0.335	4.817 ± 0.290	4.786 ± 0.069
λ (h)	4.841 ± 2.577	1.750 ± 1.898	11.280 ± 1.212
μ_max_ (Log CFU/mL/h)	−0.330 ± 0.116	−0.317 ± 0.061	0.232 ± 0.047
Final Values (Log CFU/mL)	−0.532 ± 0.357	−0.509 ± 0.230	7.548 ± 0.146
R^2^value	0.961	0.978	0.986
Standard Error of Fit	0.444	0.316	0.146
*C. botulinum*			
Initial values (Log CFU/mL)	4.165 ± 0.104	4.415 ± 0.374	4.097 ± 0.071
λ (h)	6.500 ± 0.828	4.237 ± 2.475	4.348 ± 0.917
μ_max_ (Log CFU/mL/h)	−0.387 ± 0.059	−0.360 ± 0.119	0.161 ± 0.009
Final Values (Log CFU/mL)	−0.503 ± 0.116	−0.502 ± 0.344	7.584 ± 0.094
R^2^value	0.994	0.947	0.995
Standard Error of Fit	0.162	0.482	0.094
*C. difficile*			
Initial values (Log CFU/mL)	4.535 ± 0.079	4.604 ± 0.258	4.689 ± 0.078
λ (h)	5.627 ± 0.617	3.896 ± 1.481	9.813 ± 1.361
μ_max_ (Log CFU/mL/h)	−0.351 ± 0.034	−0.395 ± 0.076	0.194 ± 0.027
Final Values (Log CFU/mL)	−0.514 ± 0.083	−0.500 ± 0.232	7.550 ± 0.153
R^2^ value	0.997	0.977	0.985
Standard Error of Fit	0.111	0.327	0.154

λ = lag phase; μmax = maximum growth rate; SPEA = saffron petals extract obtained by maceration under magnetic stirring (400 rpm); SPEB= saffron petals extract B obtained by under ultrasonic bath; CTRL = growth control.

## Data Availability

Data are available from the authors.
